# Risk Factors for Carbapenemase-Producing *Enterobacterales* Infection or Colonization in a Korean Intensive Care Unit: A Case–Control Study

**DOI:** 10.3390/antibiotics9100680

**Published:** 2020-10-08

**Authors:** Young Ah Kim, Se Ju Lee, Yoon Soo Park, Yeo Jin Lee, Jeong Hwa Yeon, Young Hee Seo, Kyungwon Lee

**Affiliations:** 1 Department of Laboratory Medicine, National Health Insurance Service Ilsan Hospital, Goyang 10444, Korea; yakim@nhimc.or.kr; 2 Department of Internal Medicine, Yonsei University College of Medicine, Seoul 03722, Korea; playit@nate.com; 3 Department of Internal Medicine, Yongin Severance Hospital, Yonsei University College of Medicine, Yongin 16995, Korea; 4 Department of Internal Medicine, National Health Insurance Service, Ilsan Hospital, Goyang 10444, Korea; 5 Infection Control Unit, National Health Insurance Service Ilsan Hospital, Goyang 10444, Korea; blue3577@nhimc.or.kr (Y.J.L.); yeonjh@nhimc.or.kr (J.H.Y.); 6 Research Institute of Bacterial Resistance, Yonsei University College of Medicine, Seoul 03722, Korea; heeyae21@hanmail.net (Y.H.S.); LEEKCP@yuhs.ac (K.L.); 7 Department of Laboratory Medicine, Yonsei University College of Medicine, Seoul 03722, Korea

**Keywords:** carbapenemase-producing *Enterobacterales*, risk factor, active surveillance culture

## Abstract

The purpose of this study is to identify the factors related to the infection and/or colonization of carbapenemase-producing *Enterobacterales* (CPE) based on clinical and microbiological data for patients in the intensive care unit (ICU). All patients admitted to medical ICU were screened for CPE on admission and weekly, and this 1:2 case–control study included patients with CPE identified by screening or clinical cultures from 2017 to 2018. The clonal relatedness was evaluated by pulsed-field gel electrophoresis (PFGE). A total of 45 CPE patients were identified with a prevalence of 3.8%. The most frequent organism was *Klebsiella pneumoniae* (69%) and the carbapenemases belonged to the class A *Klebsiella pneumoniae* Carbapenemase (KPC-2) (87%), class B New Delhi Metallo-β-lactamase (NDM) (11%), and Imipenemase (IMP-1) (2%) strains. The PFGE profiles showed two large clustered groups of KPC-2-producing *K. pneumoniae*. In the multivariate analysis, pneumonia/chronic pulmonary disease, previous fluoroquinolone use, and previous use of nasogastric tube were the significant risk factors for CPE infection or colonization in ICU-admitted patients. Critical illness and underlying medical conditions such as pneumonia/chronic pulmonary disease, antimicrobial selective pressure, and the use of a medical device are identified as risk factors for CPE infection or colonization in ICU. Person to person transmission also contributed.

## 1. Introduction

The prevalence of carbapenem-resistant *Enterobacterales* (CRE) has been increasingly reported worldwide in the past 10 years, which represents a serious threat to public health [1]. Invasive CRE infections are associated with high mortality in that the rate of CRE-attributable deaths ranged from 26% to 44% and there have become serious problems with the restricted treatment options [2]. It is estimated that as many as 9300 patients in the United States each year develop CRE infections and that more than 610 deaths are associated with these infections [3]. The production of carbapenemase is associated with the resistance mechanism of CRE, facilitating the spread of the antimicrobial resistant microorganism with the genes on the transmissible plasmids [4]. Class A *Klebsiella pneumoniae* Carbapenemase (KPC)-producing carbapenemase-producing *Enterobacterales* (CPE) are the most commonly occurring CPEs in the United States. Metallo-β-lactamases (MBL)-producing CPEs have been most commonly associated with the Indian subcontinent as well as with specific countries in Europe [5]. The high cost estimates of CPE outbreaks highlight the economic burden of antimicrobial resistance [6]. In South Korea (hereafter Korea), there have been many reports of outbreaks of CPE [7,8,9]. Although the prevalence of CPE is about 1% [10,11], the identification of CPEs seems to be the tip of the iceberg for the dissemination of notorious antimicrobial resistant pathogen in clinical settings. For the control of CPE, regional epidemiologic evaluations are needed to coordinate the efforts for infection control.

The purpose of the present case–control study was to characterize the risk factors associated with infection or colonization for CPE in patients hospitalized in the intensive care unit (ICU) of a general hospital in Korea. Molecular epidemiology was also studied. 

## 2. Results

**Figure 1:**
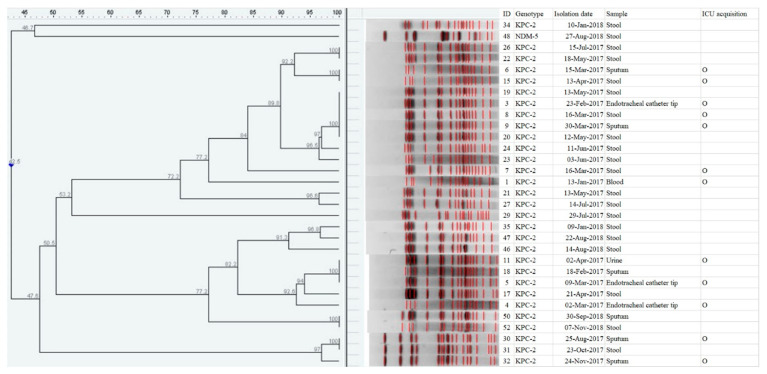
Dendrogram of *XbaI*-restricted DNA of carbapenemase-producing *Klebsiella pneumoniae* isolated from ICU-admitted patients (N = 31) (PFGE analysis was available in 31 of 32 isolates). Abbreviations: *KPC-2*, *Klebsiella pneumoniae* Carbapenemase-2; *ICU*, intensive care unit.

Of the 1176 patients admitted to the medical ICU during the study period, we identified 74 patients (6.3%) with clinical and/or surveillance cultures positive for CRE. Among them, 45 (3.8% of ICU patients) were identified as carbapenemase producers and were analyzed. Among the 45 CPE patients, 29 (65%) were identified as “influx to ICU” cases and 16 patients obtained CPE after ICU admission and were classified as “ICU acquisition” cases. According to CDC criteria, 13 patients (29%) had CPE infection, and 32 patients were colonized (Table 1). Three patients had confirmed CPE rectal colonization based on the determination of the CPE gene with Xpert Carba-R assay (Cepheid). Another 42 CPE isolates were cultured from the samples of stool (N = 29), sputum (N = 8), endotracheal catheter tip (N = 3), blood (N = 1), and urine (N = 1). The most frequent organism was *K. pneumoniae* (N = 32), followed by *Escherichia coli* (N = 5), *Enterobacter* spp. (N = 4), and *Citrobacter freundii* (N = 1). KPC-2 was the most prevalentCPE genotype (N = 39) but other types such as class B New Delhi Metallo-β-lactamase (NDM) (N = 5) and Imipenemase (IMP-1) (N = 1) were also detected. CPE isolates (N = 42) showed high antimicrobial resistance rates to ciprofloxacin (88%), cotrimoxazole (86%), tobramycin (81%), gentamicin (73.8%), colistin (62%), tetracycline (45%) in addition to carbapenem. The pulsed-field gel electrophoresis (PFGE) profiles showed two large clustered groups with 80% similarity (Figure 1).

In univariate analysis, the significant risk factor for CPE infection and/or colonization over the control was the male sex (P = 0.027), previous admission to long-term care facility (P = 0.021), pneumonia/chronic pulmonary disease (P < 0.001), previous usage of fluoroquinolone within 90 days (P < 0.001), previous history of intervention within 90 days such as urinary catheterization (P = 0.033), central catheterization (P < 0.001), intubation/tracheostomy (P = 0.006), and nasogastric tube (P < 0.001). Other factors, such as age, disease severity, and major surgery were not statistically significant (Table 2). In the multivariate analysis, pneumonia/chronic pulmonary disease (odds ratio, OR = 2.530; P = 0.045), previous use of fluoroquinolone (OR = 2.705; P = 0.035), and previous use of a nasogastric tube (OR = 10.748; P < 0.001) were found to be significant (Table 3).

## 3. Discussion

In the present study, we identified that critical illness and underlying medical conditions such as pneumonia/chronic pulmonary disease, antimicrobial selective pressure, and the use of a medical device as risk factors for CPE infection or colonization in an ICU. 

CRE causes life-threatening infections and presents high mortality rates due to the limited availability of treatment drugs. A meta-analysis of seven studies reported that 26–44% of deaths were directly related to carbapenem resistance [2]. The resistant mechanism of CRE can be largely divided into two types. The first one is the porin mutation, which inhibits the internal movement of antibacterial agents to bacteria that produce AmpC β-lactamase or extended-spectrum β-lactamase. Another is the production of carbapenemase, which has the potential to spread easily between strains via horizontal transfer due to the presence of resistant genes in mobile genetic elements. Major types of carbapenemase are class A *Klebsiella pneumoniae* Carbapenemase (KPC), class B New Delhi Metallo-β-lactamase (NDM), and class D oxacillinase [4].

CPE has been a major problem mainly in the USA, India, and Europe over the last 10 years [1]. The current prevalence of CPE is one percent on the basis of the report from the Korean Global Antimicrobial Resistance Surveillance System (Kor-GLASS), a nationwide monitoring system implemented by the Korea Centers for Disease Control and Prevention (KCDC) [11]. However, there are many reports of cases of CPE outbreaks at large hospitals [7,8,9]. The CPE National Sample Monitoring, conducted by the KCDC since 2010, has witnessed an explosive increase in reported cases of CPE. In the present study, we report a 3.8% prevalence of CPE among patients in an ICU, which is much higher than reports from Kor-GLASS, because the population in the present study comprised of patients admitted in ICU. The prevalence of this study is similar or lower compared to other regional reports: 12% in Israel, 5.4% in the USA, 6.6% in China, 12.2% in Japan, and 18.1% in India [12,13,14,15,16].

According to Kor-GLASS data of 2016, *K. pneumoniae* was the most common of all collected CPEs with a prevalence of 83.2%, and the carbapenemases were detected in *E. coli, Enterobacter* spp., *Citrobacter* spp., *Serratia marcescens*, and *Klebsiella oxytoca*. The most common genotype was the KPC, which accounts for 70.7% (N = 1029) of collected CPEs, followed by NDM (N = 197, 13.5%), oxacillinase-48 (OXA-48, N = 139, 9.6%), GES (N = 45, 3.1%), Verona Integron-encoded Metallo β-lactamase (VIM, N = 29, 2.0%), and Imipenemase (IMP, N = 16, 1.1%) [10]. In the present study, *K. pneumoniae* was the most common *Enterobacterales*, and KPC-2 was the most common genotype that was compatible with data from Kor-GLASS. In particular they showed high resistance rates to colistin in addition to carbapenemase, which could seriously limit the treatment options. 

According to the previous study, five university hospitals showed prevalence and a wide variation of dominant genotypes from institution to institution. When the clinical characteristics of CPE patients were compared with types of carbapenemase, there was no difference between patients with KPC-2-producers and patients with OXA-232-producers such as age, sex, clinical samples, ICU admission, hospital stay after CPE isolation, and mortality during hospitalization. However, the median number of hospitalization days before CPE isolation in patients with KPC-2 and OXA-232 isolates was 40 and 16 days (P = 0.006), respectively [17]. These results suggest that the CPE trend may be associated with unique characteristics of medical institutions and genotypes of carbapenemase. 

In previous studies, medical devices, antibiotics exposure, underlying disease or condition, and mechanical ventilation have been identified as risk factors of CRE [18]. In this study, pneumonia/chronic pulmonary disease, previous use of fluoroquinolone, and nasogastric tube were documented as risk factors. These results are consistent with previous reports [13,14,15,16,19,20]. Pneumonia/chronic pulmonary disease may be associated with CPE colonization or infection in the lung. A total of 11 CPE (eight from sputum, three from endotracheal catheter tip) were isolated from respiratory specimens. Mechanical ventilation was not significant risk factor in the present study, although mechanical ventilation has been documented as risk factor of CPE infection or colonization [20,21]. Carbapenem exposure and cephalosporin exposure have been the most frequently mentioned risk factors associated with CRE acquisition [18]. In present study, quinolone use was documented as a risk factor of CPE colonization or infection, consistent with previous studies [22,23]. 

The PFGE profiles showed two large clustered groups with 80% similarity. It is hypothesized that there might be person to person transmission between patients with pneumonia/chronic pulmonary disease, especially due to use of the nasogastric tube, based on the fact that pneumonia/chronic pulmonary disease and nasogastric tube were documented as risk factors and clonality was observed by PFGE. This finding suggests that during the study period, patient to patient transmission contributes to CPE infection and/or colonization with antimicrobial selective pressure. 

For CPE infection control, the detection of non-symptomatic carriers is an important factor along with passive surveillance of bacteria separated from the culture of clinical specimens. In accordance with the infection control guidelines of our health facility, CPE colonization was screened for within 48 h of admission of the patients to the ICU. Furthermore, the infection control practices were enforced at the medical ICU at the time of CPE isolation from the clinical specimen. In this study, among 45 ICU patients colonized with CPE, 29 (65%) cases were detected at the time of admission. Without active surveillance, unrecognized influx may contribute to spread of CPE in the ICU. 

We combined infection and colonization as one variable because variables were similar between infected and colonized patients, except for the Charlson co-morbidity index and long-term care facility admission history (Table 1). Most patients were colonized in the gastrointestinal tract with genetically similar antibiotic-resistant strains preceding their infection, and risk factors of infection and colonization are likely to be similar [21,24].

This study has several limitations. We did not screen for all known carbapenemases, for example GES (Guiana extended spectrum). Moreover, the small number of patients included may limit the generalization of the results. Second, as CPE isolates were obtained from both sterile and non-sterile sites, it could be confusing to differentiate between colonization and infection, despite the CDC criteria. Third, since Xpert Carba-R, a sensitive test for CPE, was not performed in all ICU patients, there was a possibility of a misclassification bias that CPE patients could be included in the control group. However, this type of misclassification only serves to underestimate the associations mentioned in this study, as it makes the case patient group and the control group more similar by including the case patient in the control group.

## 4. Materials and Methods 

The study was performed at the National Health Insurance Service (NHIS) Ilsan Hospital, an 816-bed general hospital with 16 beds for medical ICU from January 2017 to December 2018. According to the CPE surveillance program of the NHIS Ilsan Hospital, all patients admitted to the medical ICU were screened for CPE. Rectal swabs were collected from every patient at the time of ICU admission and every week. All the screening culture results as well as clinical cultures that grew CRE were studied. The infection or colonization was classified as “ICU acquisition” if the isolate was obtained after 48h of ICU admission, otherwise it was considered as “influx to ICU”. All blood cultures were considered representative of infection; urine, respiratory, and wound cultures assessed whether the infection was due to CPE as according to the CDC criteria [25]. Screening of rectal colonization was performed by following the protocol of the Centers for Disease Control and Prevention. Briefly, overnight enrichment of the stool sample or rectal swab in 5 mL trypticase soy broth (TSB) with 10 µg ertapenem or meropenem disk was performed, followed by subculturing of both the cultures onto a MacConkey agar plate. Identification and susceptibility testing were performed using a MicroScan WalkAway plus system (Beckman Coulter, Inc., West Sacramento, CA, USA) and a MicroScan Neg Breakpoint Combo Type 44 panel (Siemens Healthcare Diagnostics, Inc., West Sacramento, CA, USA). Antibiotic susceptibility was interpreted using the CLSI guidelines [26]. Xpert Carba-R test (Cepheid, Sunnyvale, CA, USA) was performed when CPE was suspected according to the judgment of the clinician. Multiplex real-time PCR test was performed to detect IMP-1, KPC, NDM, VIM, OXA-48, and OXA-48 variants (OXA-181 and OXA-232). All cultured isolates were completed with conventional PCR to detect 5 carbapenemase genes [27] and the sequencing of conventional PCR products was performed using the same primers. The clonal relatedness of *Klebsiella pneumoniae* isolates was evaluated by pulsed-field gel electrophoresis (PFGE), using restriction enzyme XbaI in the CHEF-DRII device (Bio-Rad, Hercules, CA, USA). The patterns were analyzed using Molecular Analyst Fingerprinting Software Ver. 3.2 (Bio-Rad) to generate a dendrogram based on the unweighted pair-group method, with an arithmetic average (UPGMA) based on Dice’s coefficient with 1% band-position tolerance and 0.5% optimization settings [28].

For risk factor analysis, an observational case–control study with a 1:2 ratio was conducted. The base population for the case–control study consisted of all adult (> 18 years) inpatients at the participating ICU. The case group was defined as patients who were admitted to a medical ICU and had CPE from surveillance, any clinical culture, and/or Xpert Carba-R assay from January 2017 to December 2018. For each case patient with CPE who was selected, two control patients were randomly chosen. Patients in the control group did not have CPE isolated during their ICU stay during the same study period. Patients who were ≤18 years old were excluded from the control. Clinical information (age, sex, long-term care facility admission, associated disease, Charlson morbidity index, previous antibiotic use, and intervention history) was collected by reviewing the medical records. The age was summarized as the median and other variables as N (%) of patients. Continuous variables, such as age, were analyzed using the Mann–Whitney U-test. Categorical variables were compared using the chi-squared test to identify independent risk factors. Odds ratio (OR) and 95% confidence interval (CI) values were calculated for binomial variables. Variables that yielded a p-value <0.05 in the univariate analysis were included in the multivariate logistic regression model using the forward stepwise (Wald) method to identify independent risk factors for CPE infection or colonization. Statistical significance was defined as p < 0.05. Data were analyzed using SPSS version 23 (IBM Corp., Armonk, NY, USA). The study was approved by institutional review boards as required by local hospital policy (NHIMC 2018-01-042).

## 5. Conclusions

In this study, pneumonia/chronic pulmonary disease, previous use of fluoroquinolone, and nasogastric tube were documented as risk factors of CPE infection or colonization in the ICU. With result of PFGE, person to person transmission of CPE also documented. These findings underscore the importance of antibiotic stewardship, for fluoroquinolone especially, in patients with pneumonia/chronic pulmonary disease and using a nasogastric tube. Contact precaution should be emphasized to prevent person to person transmission of CPE in ICUs.

## Figures and Tables

**Table 1:** Baseline characteristics of patients infected or colonized with carbapenemase-producing *Enterobacterales* in medical intensive care unit.

	Infection (N = 13)	Colonization (N = 32)	P-value
Age (years)	78 (71–85)	73 (61–83)	0.15
Male	8 (62)	25 (78)	0.285
Previous admission to long-term care facility	8 (62)	9 (28)	0.048
Associated disease			
End-stage renal disease	4 (31)	4 (13)	0.202
Gastrointestinal disease	2 (15)	6 (19)	1
Cerebrovascular disease	3 (23)	5 (16)	0.672
Localized infection	2 (15)	4 (13)	1
Pneumonia/chronic pulmonary disease	10 (77)	21 (66)	0.724
Diabetes mellitus	3 (23)	5 (16)	0.672
Cardiovascular disease	2 (15)	3 (9)	0.617
Malignancy	3 (23)	1 (3)	0.066
Sepsis	3 (23)	7 (22)	1
Charlson comorbidity index	6 (6–8)	5 (4–6)	0.019
Previous usage of antibiotics within 90 days			
Penicillin	9 (69)	18 (56)	0.514
Fluoroquinolone	8 (62)	17 (53)	0.745
Carbapenem	7 (54)	12 (38)	0.341
1st generation cephalosporin	0	1 (3)	
2nd generation cephalosporin	0	2 (6)	
Expanded-spectrum cephalosporin	4 (31)	9 (28)	1
Glycopeptide	7 (54)	14 (44)	0.743
Macrolide	0	2 (6)	1
Previous history of intervention within 90 days			
Urinary catheterization	13 (100)	30 (94)	1
Central catheterization	12 (92)	28 (88)	1
Intubation/tracheostomy	9 (69)	19 (59)	0.737
Nasogastric tube	12 (92)	29 (91)	0.546
Major surgery	2 (15)	3 (9)	0.617

Number (%) or Median (interquartile range).

**Table 2:** Univariate analysis of risk factors for infection or colonization of carbapenemase-producing *Enterobacterales* in medical intensive care unit using univariate analysis.

Head	Controls (N = 90)	Cases (N = 45)	OR (95% CI)	P-value
Age (years)	76 (59–82)	76 (63–84)		0.330
**Male**	**48 (53)**	**33 (73)**	**2.4 (1.1–5.2)**	**0.027**
**Previous admission to long-term care facility**	**17 (19)**	**17 (38)**	**2.6 (1.2–5.8)**	**0.021**
Associated disease				
End-stage renal disease	14 (16)	8 (18)	1.2 (0.5–3.0)	0.806
Gastrointestinal disease	14 (16)	8 (18)	1.2 (0.5–3.0)	0.806
Cerebrovascular disease	21 (23)	8 (18)	0.7 (0.3–1.8)	0.512
Localized infection	6 (7)	6 (13)	2.2 (0.7–7.1)	0.214
**Pneumonia/chronic pulmonary disease**	**29 (32)**	**31 (69)**	**4.7 (2.2–10.0)**	**<0.001**
Diabetes mellitus	17 (19)	8 (18)	0.9 (0.4–2.4)	1
Cardiovascular disease	14 (16)	5 (11)	0.7 (0.2–2.0)	0.604
Malignancy	10 (11)	4 (9)	0.8 (0.2–2.6)	0.774
Sepsis	15 (17)	10 (22)	1.4 (0.6–3.5)	0.484
Charlson comorbidity index	5 [4,5,6,7]	6 [5,6,7]		0.526
Previous usage of antibiotics within 90 days				
Penicillin	47 (52)	27 (60)	1.4 (0.7–2.8)	0.464
**Fluoroquinolone**	**19 (21)**	**25 (56)**	**4.7 (2.2–10.1)**	**<0.001**
Carbapenem	30 (33)	19 (42)	1.5 (0.7–3.1)	0.346
1st generation cephalosporin	6 (7)	1 (2)	0.3 (0.04–2.7)	0.424
2nd generation cephalosporin	3 (3)	2 (4)	1.3 (0.2–8.4)	1
Expanded-spectrum cephalosporin	41 (46)	13 (29)	0.5 (0.2–1.0)	0.066
Glycopeptide	34 (38)	21 (47)	1.4 (0.7–3.0)	0.356
Macrolide	7 (8)	2 (4)	0.6 (0.1–2.8)	0.717
Previous history of intervention within 90 days				
**Urinary catheterization**	**73 (81)**	**43 (96)**	**5.0 (1.1–22.7)**	**0.033**
**Central catheterization**	**50 (56)**	**40 (89)**	**6.4 (2.3–17.7)**	**<0.001**
**Intubation/tracheostomy**	**32 (36)**	**28 (62)**	**3.0 (1.4–6.3)**	**0.006**
**Nasogastric tube**	**46 (51)**	**42 (93)**	**13.4 (3.9–46.4)**	**<0.001**
Major surgery	4 (4)	5 (11)	2.7 (0.7–10.5)	0.160

Number (%) or Median (interquartile range); CI, confidence interval; OR, odds ratio; Bold formatting indicates statistical significance.

**Table 3:** Risk factors for infection and/or colonization of carbapenemase-producing *Enterobacterales* in medical intensive care unit using multivariate analysis.

Head	OR (95% CI)	P-value
Pneumonia/chronic pulmonary disease	2.5 (1.02–6.3)	0.045
Previous use of fluoroquinolone	2.7 (1.07–6.8)	0.035
Previous use of nasogastric tube	10.7 (3.0–38.7)	< 0.001

CI, confidence interval; OR, odds ratio; bold formatting indicates statistical significance.
